# Supplementary material for: Postmitotic differentiation of human monocytes requires cohesin-structured chromatin
Source: Nat Commun. 2022 Jul 25;13:4301. doi: 10.1038/s41467-022-31892-2 (PMC9314343; doi:10.1038/s41467-022-31892-2)
Supplement: Supplementary file 1 — Supplementary Information [file 41467_2022_31892_MOESM1_ESM.pdf]

Supplementary Information for

Postmitotic differentiation of human monocytes requires cohesin-structured chromatin

Julia Minderjahn,[1,5] Alexander Fischer,[1] Konstantin Maier,[1] Karina Mendes,[1,6] Ute Ackermann,[1] Margit Nuetzel,[1] Johanna Raithel,[2] Hanna Stanewsky,[1] Robert Månsson,[3] Claudia Gebhard,[2] Michael Rehli[1,2,4,*]

[1]Department of Internal Medicine III, University Hospital Regensburg, 93053 Regensburg, Germany
[2]Leibniz Institute for Immunotherapy, c/o University Hospital Regensburg, 93053 Regensburg, Germany
[3]Center for Hematology and Regenerative Medicine Huddinge, Karolinska Institutet, Stockholm, Sweden

[4]Lead Contact
[*]Correspondence: michael.rehli@ukr.de

[5]present address: Sandoz GmbH, Biochemiestraße 10, 6336 Langkampfen, Austria
[6]present address: Universidade Católica Portuguesa, Center for Interdisciplinary Research in Health (CIIS), Institute of Health Sciences (ICS), Viseu, Portugal

Supplement Index:

Supplementary Figures 1-9 page 02-13
Supplementary Tables 1-15 page 14-26
Supplementary References page 27

Supplementary Figures & Legends

Supplementary Figure 1

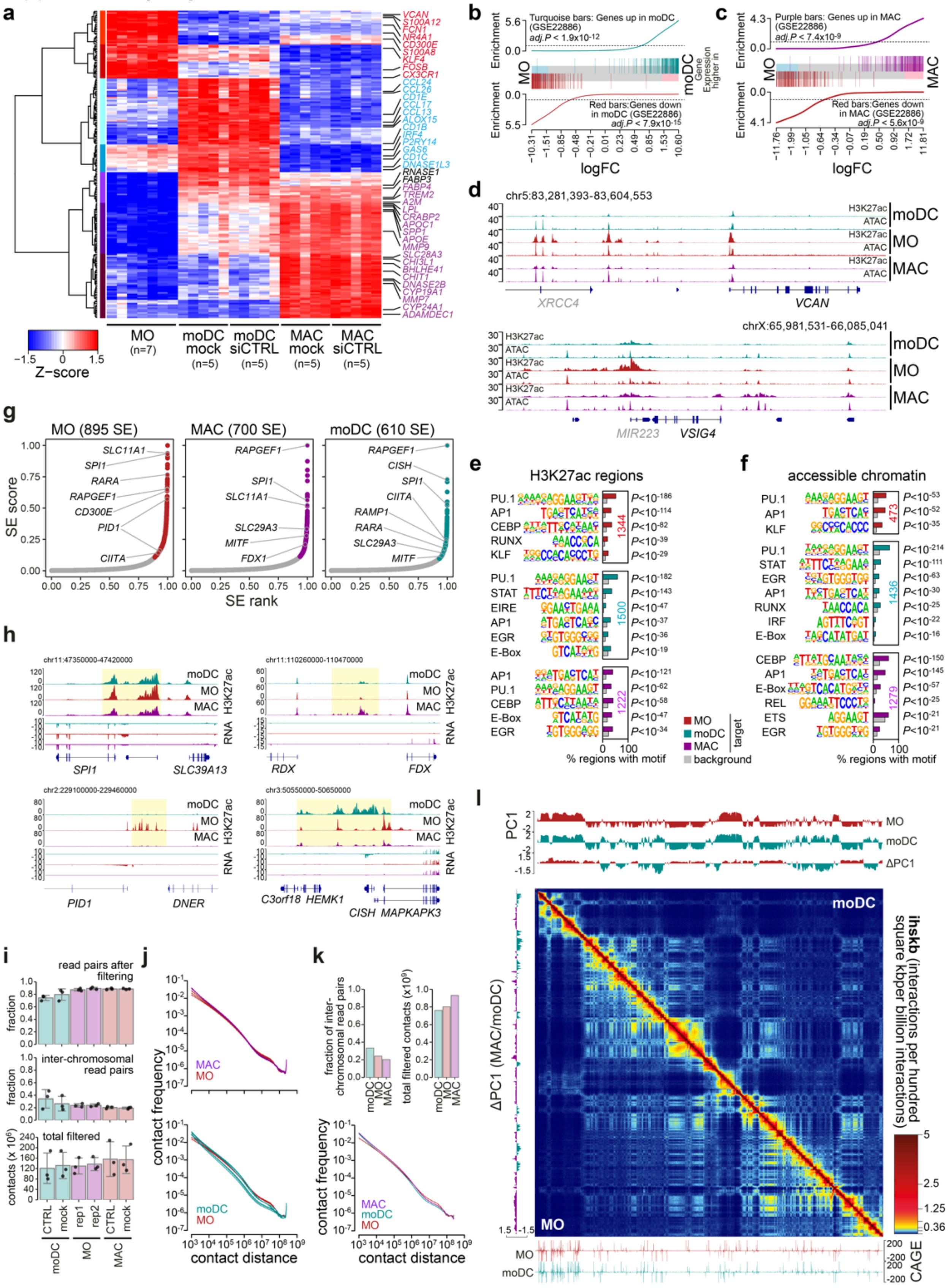

(Continued on next page)

Epigenetic, transcriptional and architectural changes during MO differentiation, Related to Figure 1. a Heatmap presenting hierarchically clustered and scaled expression data of the top 200 variable expressed genes across MO, moDC and MAC data sets. Each column corresponds to an individual donor. Known marker genes are indicated and colored corresponding to the cell type with the highest expression (MO, red; moDC, turquoise; MAC, purple). **b,c** Bar code plots showing results for the top gene set enriched in MO/moDC (in **b**) or MO/MAC comparisons (in **c**). Enrichment of genes associated with the indicated gene sets (MO in red, MAC in purple, moDC in turquoise) is shown across all genes ranked by their expression in the indicated cell types. *P*-values were determined using two-sided competitive tests and BH corrected. **d** Genome browser tracks for the indicated MO- and MAC-specific example regions. **e,f** *De novo* identified sequence motifs across H3K27ac-marked accessible regions (in **e**) or all accessible regions (in **f**) specific for MO, moDC or MAC. Motif enrichment is indicated by bar plots, along with enrichment *P* values (derived from hypergeometric tests). **g** Hockey stick plots identifying super enhancers (SE) based on input-normalized H3K27ac ChIP-seq signals (SE scores). SEs (inflection value ≥ 1) are indicated by colored dots.**h** Genome browser tracks for SE example regions. **i-k** Quality controls for Hi-C experiments (n=3 independent donors). In **i**, the fractions of unique read pairs after filtering of local interactions (<1kb) for individual settings and replicates (top panel), fractions of interchromosomal read pairs (mid panel) and total contacts after filtering (bottom panel) are shown (data are presented as mean values +/- SD). In **j**, histograms describing the fraction of paired-end reads that are found at different distances from one another (bin size: 1kb). In **k**, metrics for the merged data of the three cell types are shown. **l** *In situ* Hi-C contact map of primary MO (lower left) and moDC (upper right) across a 50Mb interval of chromosome 1. Map represents the average of 3 (MO) or 6 replicates (moDC) per condition. Top, PC1 values (compartments), including difference tracks as indicated. Bottom, CAGE-seq data. (a-c,e,f,i,k) Source data are provided as a Source Data file.

Supplementary Figure 2

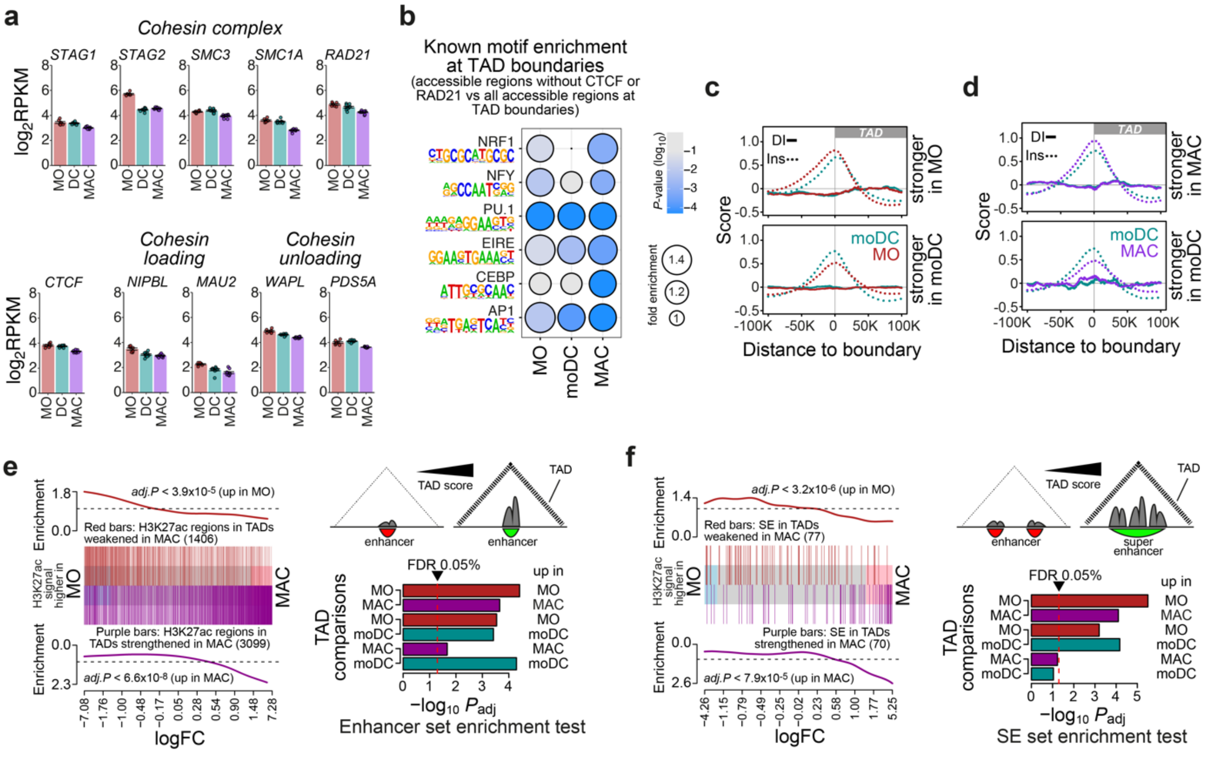

Changes in topology-associated domains (TADs) during MO differentiation correlate with gene expression changes, Related to Figure 2. a Bar plots showing expression levels (mean ± SE based on the RNaseq data; n=6 control samples from three donors) of cohesin-associated genes. **b** Motif enrichment across open chromatin regions overlapping TAD boundaries that lack CTCF or RAD21 peaks. Known motifs were selected from *de novo*-analyses in individual samples and enriched motifs correspond to known enhancer signature motifs in MO, MAC and moDC (PU.1, C/EBP, AP-1, EIRE), or general house-keeping promoter motifs (NRF1, NF-Y). *P*-values were determined using hypergeometric tests and adjusted for multiple testing (BH correction). **c,d** Histograms of insulation scores (Ins, dotted lines) and directionality indices (indicating preferences for contacts either upstream or downstream) across differential TAD boundaries for MO versus moDC (in **c**) or moDC versus MAC comparisons (in **d**). **e** Peak set enrichment of differential TAD-associated H3K27ac peak regions. The enrichment of peaks associated with cell type-specific TADs (MO in red, MAC in purple) across all peaks ranked by their signal in MO versus MAC is shown exemplary in the left panel. Results of peak set enrichment testing for all MO, MAC, moDC comparisons are summarized in the bar plot shown in the bottom right panel, indicating that changes in TAD strengths tend to associate with accordant changes in signals of TAD-overlapping H3K27ac peak regions, as depicted in the schematic on top of the bar plot. **f** Peak set enrichment of differential TAD-associated super-enhancer (SE) regions. The enrichment of SEs associated with cell type-specific TADs (MO in red, MAC in purple) across all peaks ranked by their signal in MO versus MAC is shown exemplary in the left panel. Results of peak set enrichment testing for all MO, MAC, moDC comparisons are summarized in the bar plot shown in the bottom right panel, indicating that changes in TAD strengths tend to associate with accordant changes in signals of TAD-overlapping H3K27ac peak regions (except for the MAC-moDC comparison), as depicted in the schematic on top of the bar plot. *P*-values in (**e,f**) were determined using two-sided rotation tests and adjusted for multiple testing (BH correction). (a-f) Source data are provided as a Source Data file.

Supplementary Figure 3

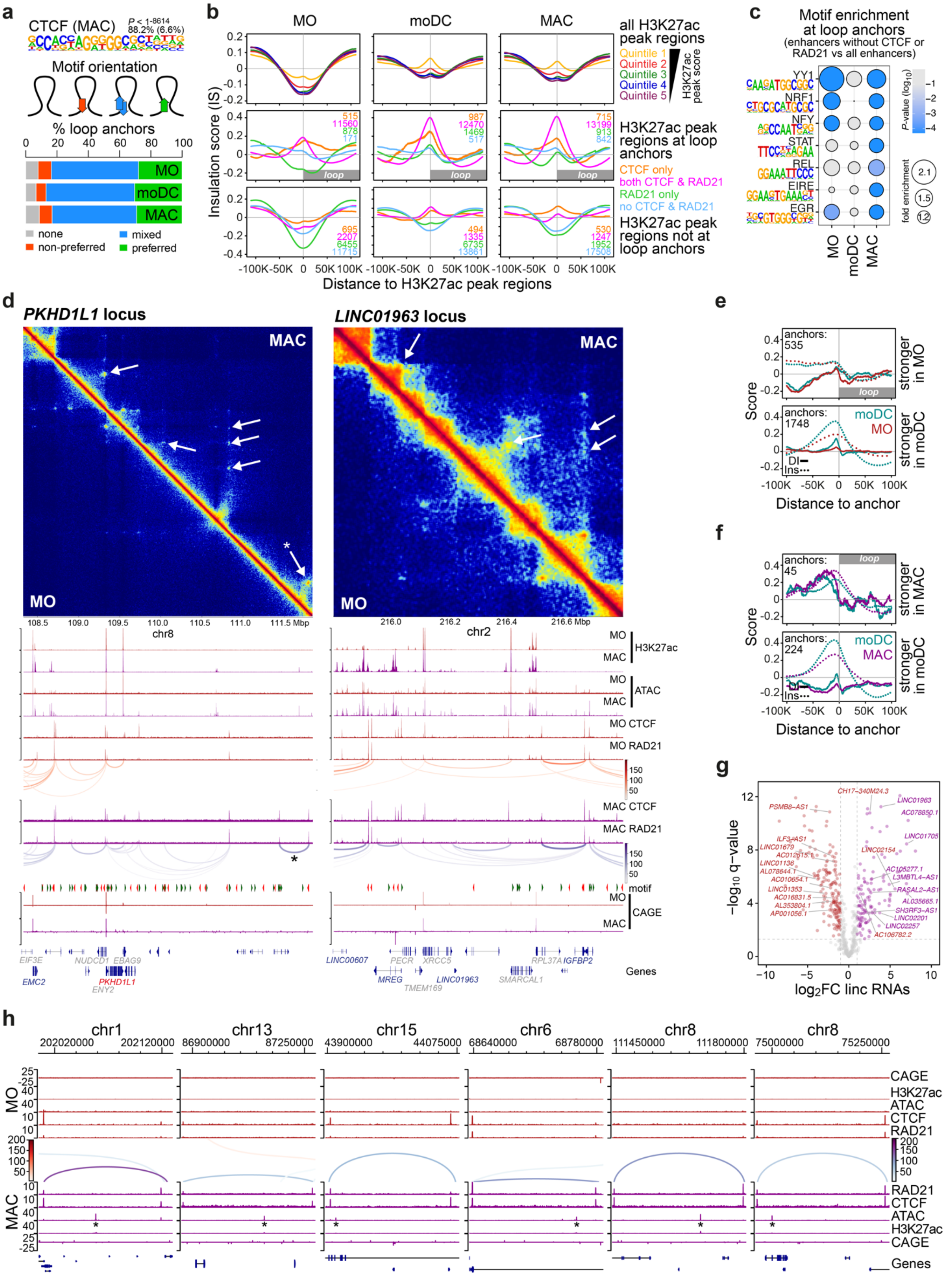

(Continued on next page)

Changes in chromatin loops during MO differentiation correlate with gene expression changes, Related to Figure 3. **a** Association of loop anchors with CTCF motifs (MAC *de novo*-derived CTCF motif used for mapping is shown on top, *P* value: hypergeometric test). Most loop anchors contain at least one motif facing in the preferred direction (blue and green bars). **b** Histograms of insulation scores H3K27ac peak sets in MO, moDC and MAC. Top panel, H3K27ac peaks binned according to signal strength; central panel, H3K27ac peaks overlapping loop anchors depending on their overlap with CTCF and RAD21 peaks; bottom panel, H3K27ac peaks not overlapping loop anchors depending on their overlap with CTCF and RAD21 peaks **c** Motif enrichment across open chromatin associated with H3K27ac peak regions overlapping loop anchors that lack CTCF or RAD21 peaks (using all loop associated H3K27ac regions as background). Known motifs were selected from *de novo*-analyses in individual samples and enriched motifs correspond to known motifs in MO, MAC and moDC associated with signalling events (STAT, REL, EIRE, EGR), or general house-keeping promoter motifs (YY1, NRF1, NF-Y). *P*-values were determined using hypergeometric tests and BH corrected. **d** Additional examples of comparative *in situ* Hi-C interaction maps and corresponding genome browser tracks for indicated example regions comparing MO and MAC. Tracks include ATAC-seq, ChIP-seq (H3K27ac, RAD21, CTCF) and CAGE-seq data as indicated. **e,f** Histograms of insulation scores (Ins, dotted lines) and directionality indices (indicating preferences for contacts either upstream or downstream) across differential loop anchors for MO versus moDC (in **e**) or moDC versus MAC comparisons (in **f**). **g** Volcano plot focusing on lincRNA genes and highlighting differentially expressed lincRNAs in MO (red dots) vs. MAC (purple dots). Loop associated lincRNA genes are marked by their gene symbols and colors indicate their association with MO-enriched (red lettering) or MAC-enriched loops (purple lettering). **h** Genome tracks for examples of cell type-specific loop formation. (a-c,e-g) Source data are provided as a Source Data file.

Supplementary Figure 4

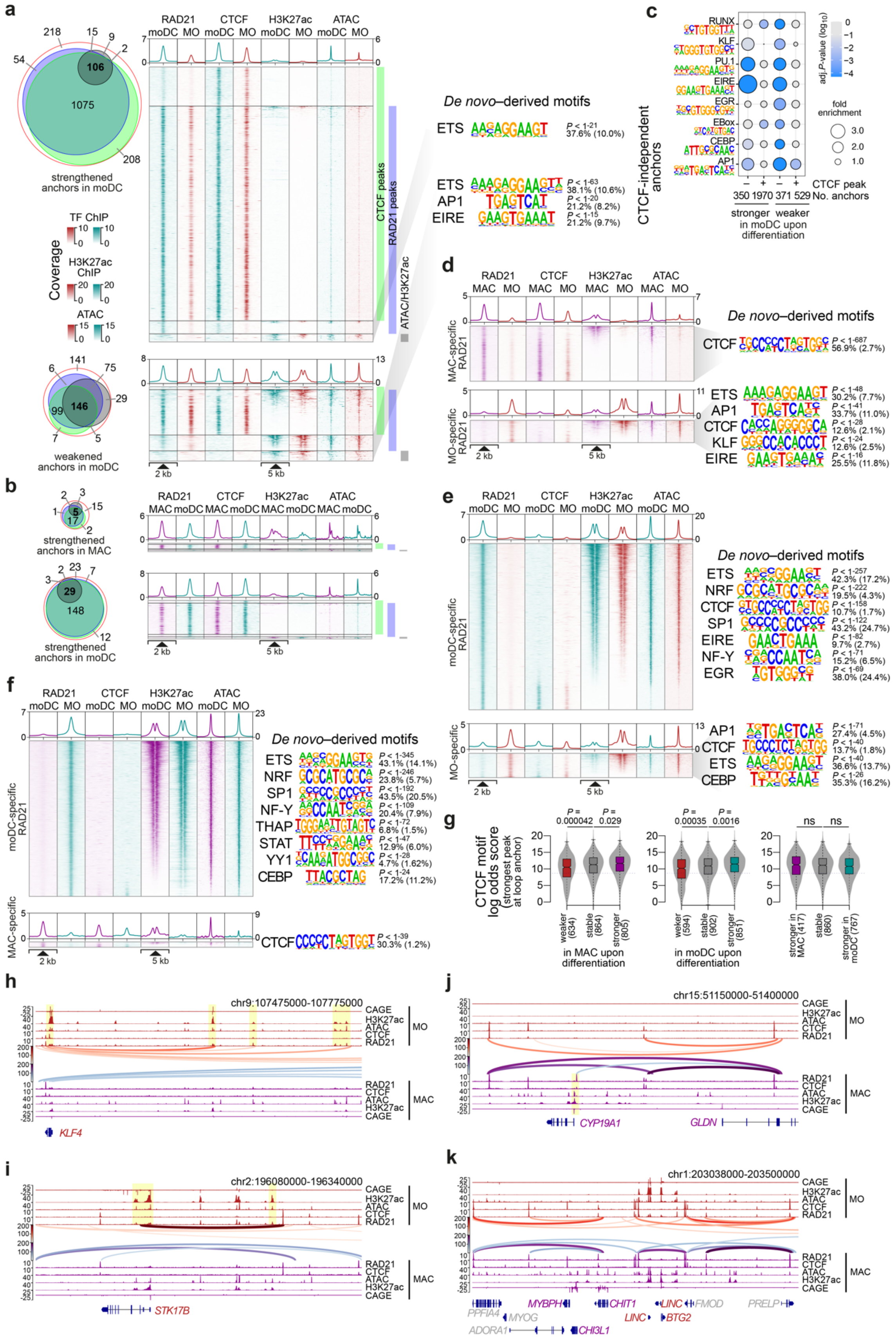

(Continued on next page)

Differentiation-associated shift from regulatory to structural loops, Related to Figure 4. a,b Genomic distance distribution of average signals for RAD21, CTCF, H3K27ac and ATAC sequencing data across peak-centered loop anchors that are either strengthened or weakened during MO differentiation into moDC (in a) or in moDC as compared to MAC (in b), as described in Figure 4a for MAC. Venn diagrams on the left present the overlap between cell type-specific loop anchors, RAD21, CTCF and H3K27ac peaks for the given comparisons. Top *de novo*-derived motifs for CTCF-independent loop anchors are given on the right along with the significance of motif enrichment (*P* values: hypergeometric test, BH adjusted) and the fraction of motifs in peaks (background values are in parenthesis). **c** Motif enrichment across CTCF-overlapping and non-overlapping differential loop anchors compared to a random genomic background. The motif search space was either confined to open chromatin regions (no CTCF overlap) or CTCF peaks regions overlapping loop anchors. Known motifs were selected from *de novo*-analyses in individual cell types (*P* values are from hypergeometric tests, BH adjusted). **d-f** Genomic distance distribution of average signals for RAD21, CTCF, H3K27ac and ATAC sequencing data across RAD21 peaks that were either stronger or weaker (at least three-fold) in the indicated comparisons. Motifs discovered *de novo* in each differential peak set are displayed as in a (*P* values: hypergeometric test, BH adjusted). **g** CTCF motif score distribution across peaks within the indicated loop anchors (*P* values: Mann–Whitney U-test, two-sided). **h-k** Genome tracks showing example regions harboring differentiation-associated regulatory and structural loops. (a-g) Source data are provided as a Source Data file.

Supplementary Figure 5

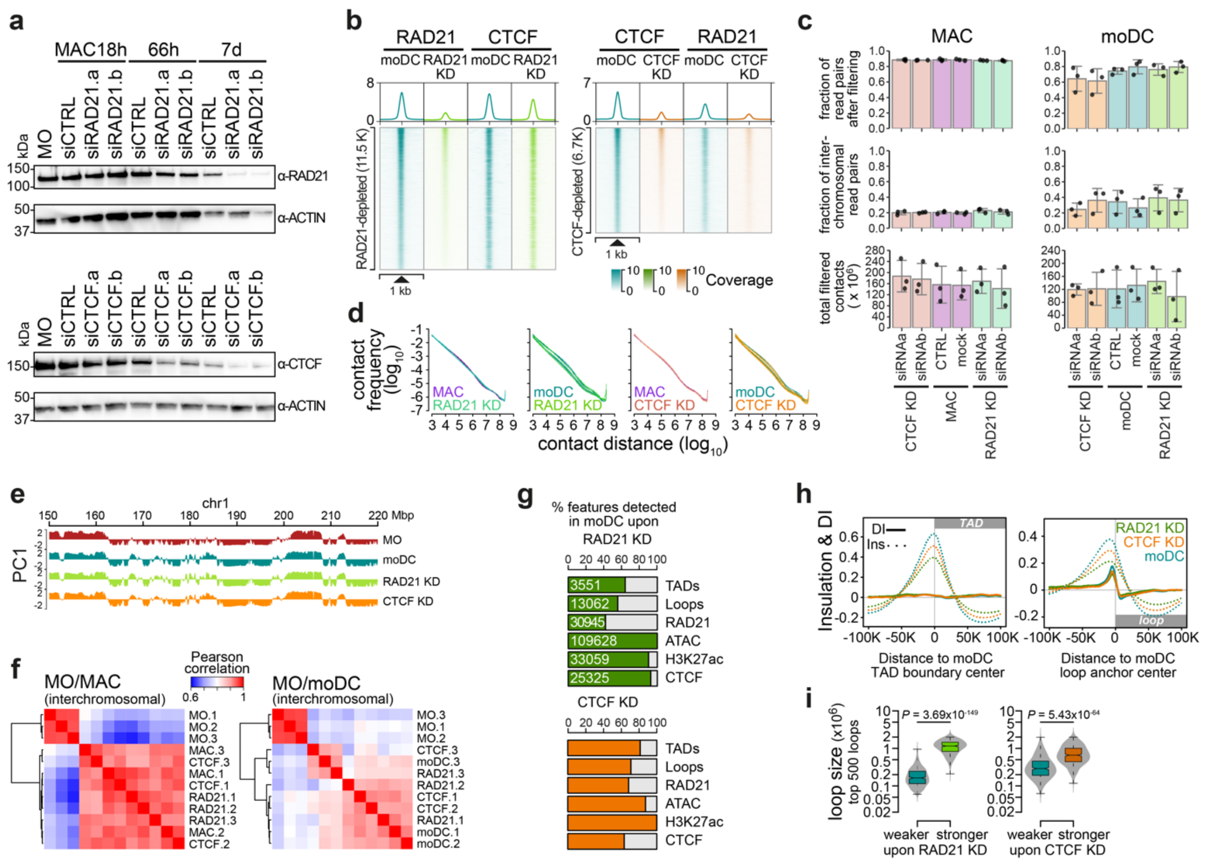

Cohesin knockdown significantly affects 3D genome architecture during MO differentiation, Related to Figure 5. **a** RAD21 and CTCF protein expression levels in MO, and siRNA or siCTRL-treated MAC at the indicated time points (18h, 66h and 7d). Blots were stained with α-CTCF, α-RAD21 and α-ACTIN antibodies (the latter represents the protein loading control). Shown is one representative out of two experiments. **b** Genomic distance distributions of RAD21 and CTCF ChIP-seq coverage at RAD21 peaks with significantly reduced signal in RAD21 knockdown moDC (left panel) or CTCF peaks with significantly reduced signal in CTCF knockdown moDC. **c,d** Quality controls for Hi-C experiments (n=3 independent donors). In **c**, the fractions of unique read pairs after filtering of local interactions (<1kb) for individual settings and replicates (top panel), fractions of interchromosomal read pairs (mid panel) and total contacts after filtering (bottom panel) are shown (data are presented as mean values +/- SD). In **d**, histograms describing the fraction of paired-end reads that are found at different distances from one another (bin size: 1kb). **e** Tracks representing the first eigenvector values (PC1) of a principal component analysis (PCA) on a Hi-C correlation matrix at 50kB resolution for the indicated cell types. **f** Clustering of Pearson correlation values for all significant interchromosomal contacts between knockdown and control cells. **g** Effect of the RAD21 or CTCF knockdown on the indicated features. Colored bars represent the number of detectable features as percentage of the same feature in control moDC. Numbers represent feature counts in control moDC. **h** Histogram of insulation scores (Ins, dotted lines) and directionality indices (indicating preferences for contacts either upstream or downstream) across all loop anchor regions for siRNA-treated or control-treated moDC. **i** Distribution of loop sizes for the top 500 domains gained or lost upon RAD21 or CTCF knockdown in moDC. Solid bars of boxes represent the interquartile ranges (25–75%) with an intersection at the median; whiskers represent max/min values; *P* values: Mann–Whitney U-test, two-sided. (a,c,f,h,i) Source data are provided as a Source Data file.

Supplementary Figure 6

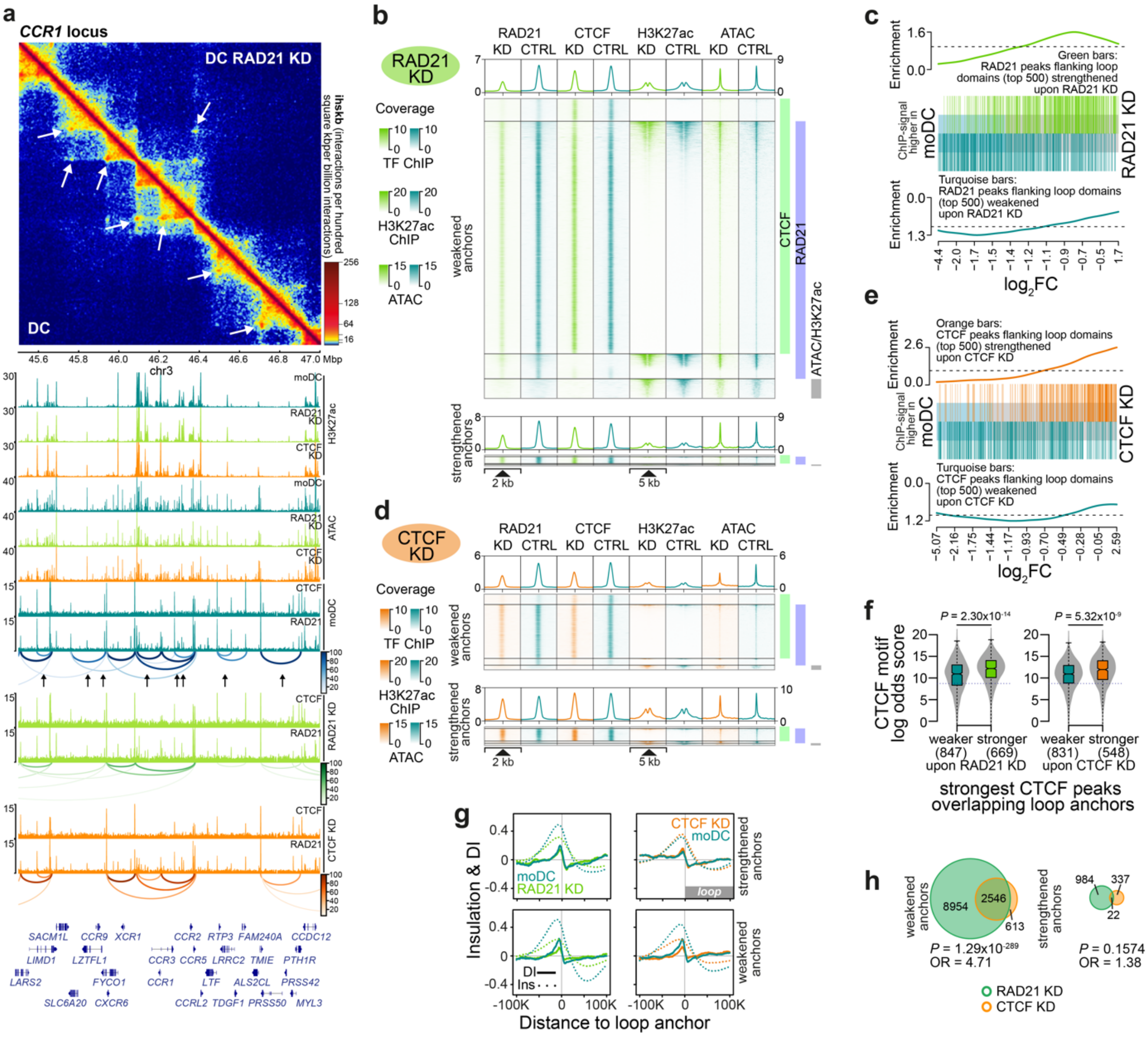

Properties of loop domain boundaries altered upon cohesin depletion, Related to Figure 6. a Comparative *in situ* Hi-C interaction map and corresponding genome browser tracks for the *CCR1* locus on Chromosome 3. Differential loops are indicated by arrows. **b** Genomic distribution of average signals for RAD21, CTCF, H3K27ac and ATAC sequencing data across peak-centered loop anchors that are either strengthened or weakened by RAD21 knockdown in moDC. **c** Peak set enrichment of loop anchor-associated RAD21 peaks. The enrichment of peaks associated with loop anchors that are either strengthened or weakened by RAD21 knockdown across all peaks ranked by their signal in siRNA-treated versus control-treated moDC is plotted. **d** Genomic distribution as described in (**b**) for the CTCF knockdown. **e** Peak set enrichment of loop anchor-associated CTCF peaks as described in (**c**) for the CTCF knockdown. **f** CTCF motif score distribution across the indicated loop anchors. Solid bars of boxes represent the interquartile ranges (25–75%) with an intersection at the median; whiskers represent max/min values; *P* values: Mann–Whitney U-test, two-sided; dotted line: detection threshold. **g** Histograms of insulation scores (Ins, dotted lines) and directionality indices across differential loop anchor regions for siRNA-treated (RAD21 KD, CTCF KD) and control-treated moDC. **h** Venn diagrams of anchors overlapping strengthened and weakened loops of both siRNA treatments. (*P* values and Odds ratios from two-sided Fisher's exact tests are given below). (b,d,f-h) Source data are provided as a Source Data file.

Supplementary Figure 7

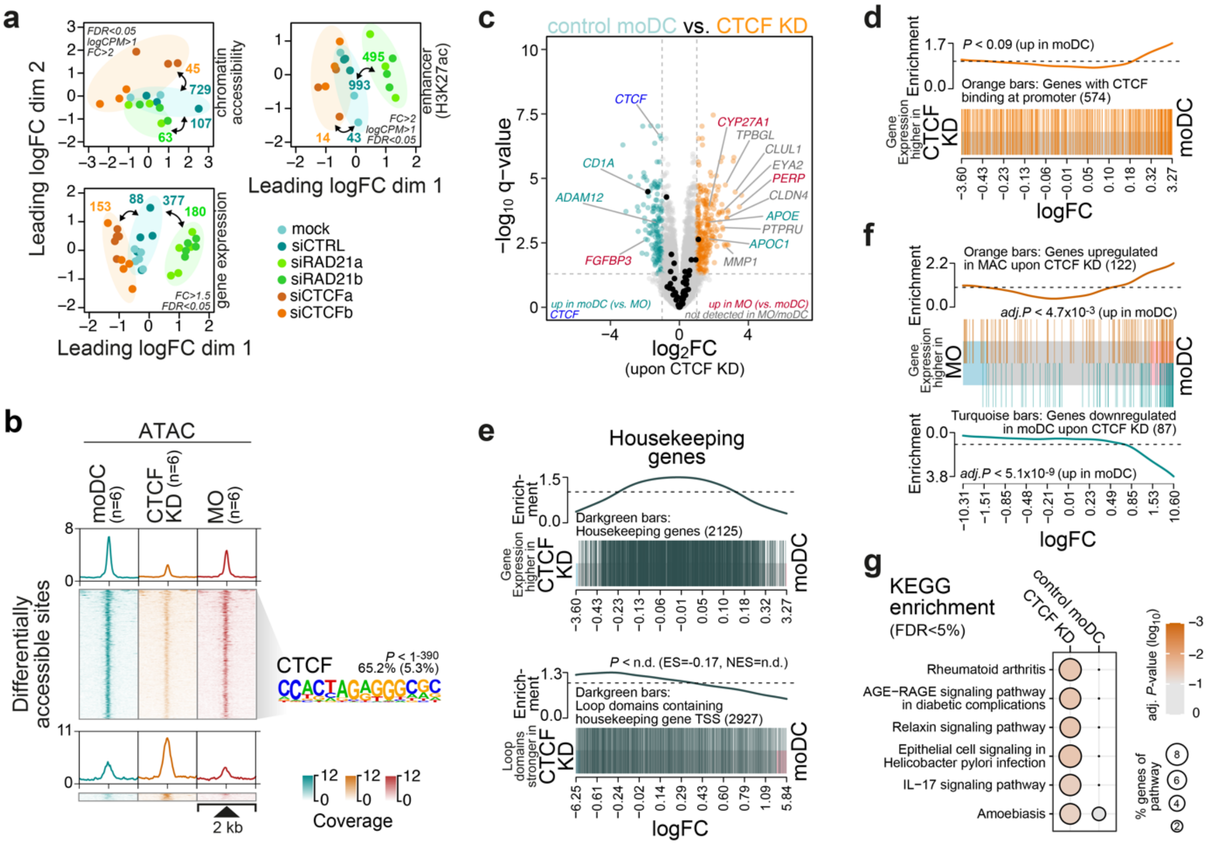

CTCF knockdown affects MO differentiation-dependent transcriptional programs, Related to Figure 7. a MDS plots of ATAC-seq, RNA-seq and H3K27ac ChIP-seq data sets for moDC control cells and siRNA knockdowns. Numbers of differentially accessible sites, expressed genes, or H3K27ac marked histones are indicated. **b** Distribution of ATAC-seq signals across differentially accessible sites in control moDC, siRNA-treated cells (RAD21 KD and CTCF KD) as well as freshly isolated MO. *De novo*-derived motifs were only available for the cluster losing accessibility upon CTCF knockdown. The motif is given along with the significance of motif enrichment (hypergeometric test) and the fraction of motifs in peaks (background values are in parenthesis). **c** Volcano plot of genes differentially expressed in CTCF knockdown moDC. Exemplary genes are highlighted. Purple dots, downregulated upon KD. Salmon dots, upregulated upon KD. Black dots, top 50 upregulated genes during normal MO differentiation. Gene symbols in gray, not detected in MO or control moDC. Gene symbols in red, genes downregulated during MO differentiation. Gene symbols in turquoise, genes upregulated during MO differentiation. **d** Enrichment of the set of genes with promoter-associated CTCF peaks across all genes ranked by their signal in CTCF siRNA-treated versus control-treated moDC (*P*-value: two-sided rotation test). **e** Analysis of housekeeping genes. Top panel: Gene set enrichment of housekeeping genes across all genes ranked by their signal in siRNA-treated versus control-treated moDC (no significant enrichment using two-sided rotation tests). Bottom panel: loop set enrichment for domains containing housekeeping genes across all loop domains ranked by their score in siRNA-treated versus control-treated moDC (ES, enrichment score; NES, normalized enrichment score; *P* not determined due to unbalanced gene-level statistics). **f** Gene set enrichment of genes that were differentially expressed upon CTCF KD across all genes ranked by their signal in MO versus moDC. (*P*-values: two-sided rotation tests, BH correction). **g** KEGG pathway analysis of genes affected by CTCF loss in moDC (*P*-values: hypergeometric tests, BH correction).. (b,d,g) Source data are provided as a Source Data file.

Supplementary Figure 8

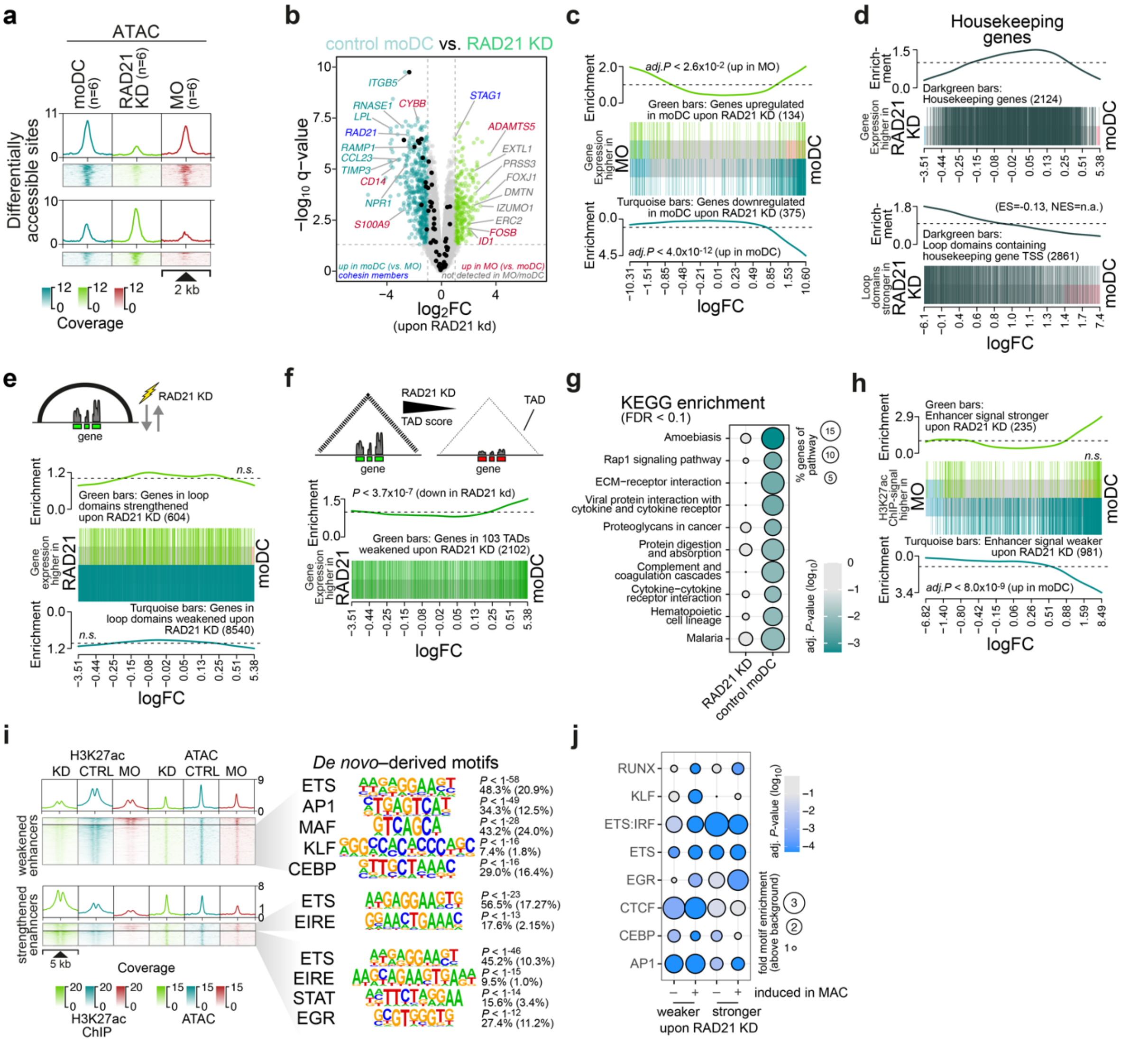

Loss of cohesin profoundly affects MO differentiation-dependent transcriptional programs, Related to Figure 8. **a** Distribution of ATAC-seq signals across differentially accessible sites in control moDC, RAD21 siRNA-treated cells as well as freshly isolated MO. Due to the low number of detected sites, *de novo* motif analyses did not reveal significantly enriched motifs. **b** Volcano plot of genes differentially expressed in control versus RAD21 knockdown moDC. Exemplary genes are highlighted. Turquoise dots, downregulated upon KD. Green dots, upregulated upon KD. Black dots, top 50 upregulated genes during normal MO differentiation. Gene symbols in gray, not detected in MO or control moDC. Gene symbols in red, genes downregulated during MO differentiation. Gene symbols in purple, genes upregulated during MO differentiation. **c** Gene set enrichment of genes that were differentially expressed upon RAD21 KD across all genes ranked by their signal in MO versus moDC. **d** Analysis of housekeeping genes. Top panel: Gene set enrichment of housekeeping genes across all genes ranked by their signal in siRNA-treated versus control-treated moDC (no significant enrichment using two-sided rotation tests). Bottom panel: loop set enrichment for domains containing housekeeping genes across all loop domains ranked by their inclusion score in siRNA-treated versus control-treated moDC (ES, enrichment score; NES, normalized enrichment score; P not determined due to unbalanced gene-level statistics). **e** Enrichment of genes in loop domains that were either strengthened (green bars) or weakened (turquoise bars) across all genes ranked by their signal in siRNA-treated versus control-treated moDC. **f** Gene set enrichment of genes in TADs that were weakened upon during MO differentiation in RAD21 knockdown moDC. **g** KEGG pathway analysis of genes affected by RAD21 loss in moDC. **h** Peak set enrichment of differentially active enhancers. Enrichment of enhancers (H3K27ac-marked regions) affected by RAD21 KD (Green, increased signal; turquoise, decreased signal) across all enhancers ranked by their signal intensity in MO versus moDC. **i** Genomic distance distributions at differentially active regulatory regions (based on their H3K27ac deposition), centered on overlapping open chromatin. *De novo*-derived motifs were only available for the cluster losing H3K27ac upon RAD21 knockdown while gaining it during MO differentiation. Motifs are given along with the significance of motif enrichment (hypergeometric test) and the fraction of motifs in peaks (background values are in parenthesis). **j** Motif enrichment across open chromatin associated with H3K27ac peak regions as shown in **i**. Known motifs were selected from *de novo*-analyses in individual samples. *P*-values were determined using hypergeometric tests in (**g,i,j**) or two-sided rotation tests in (**c,e,f,h**) and adjusted for multiple testing (BH correction) except in (**i,f**). (g,i,j) Source data are provided as a Source Data file.

Supplementary Figure 9

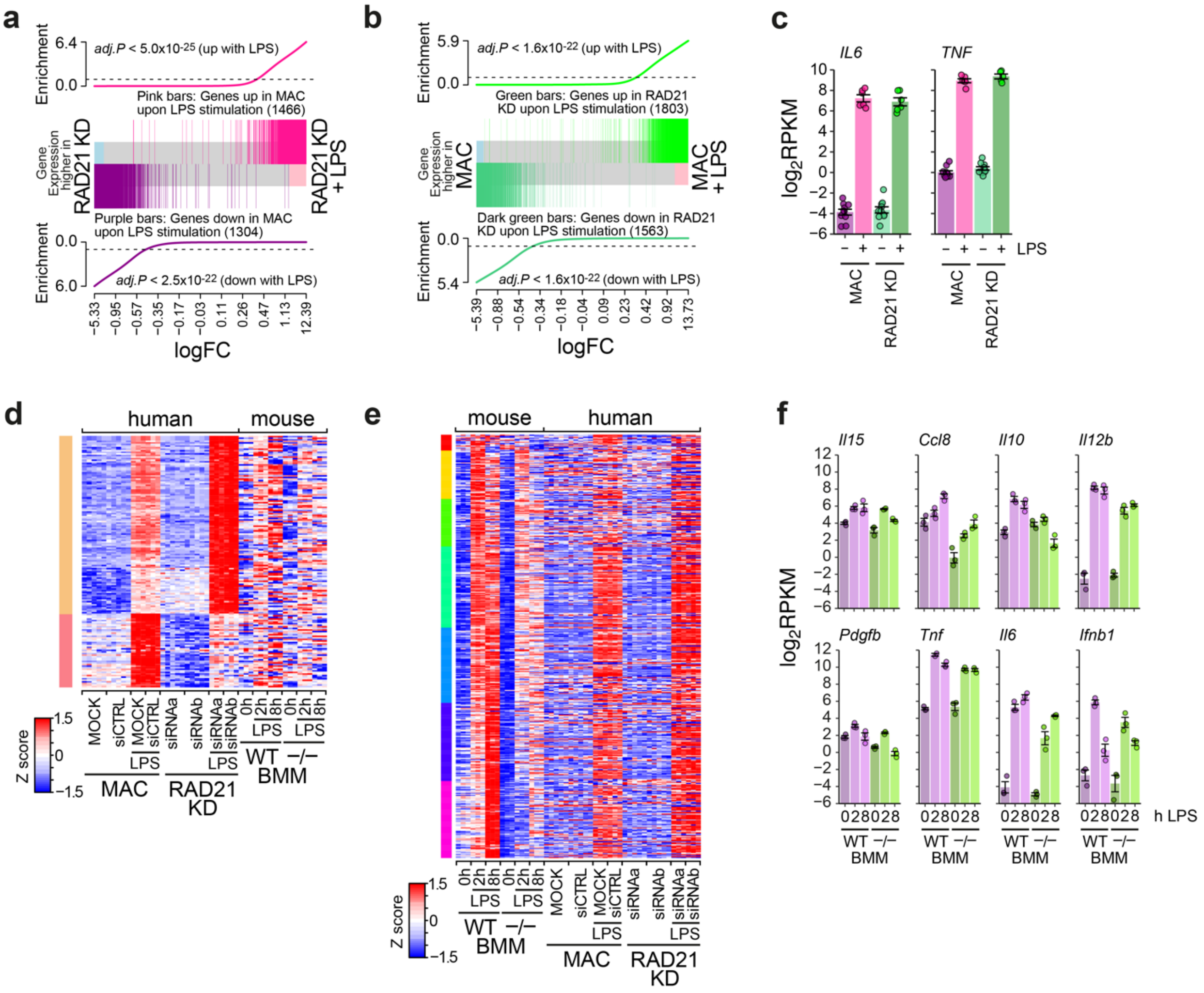

Loss of cohesin alters innate activation programs in MAC, Related to Figure 9. a Gene set enrichment of genes regulated by LPS in control MAC during LPS stimulation of RAD21 KD MAC. **b** Gene set enrichment of genes (mean normalized RPKM ± SE based on the RNA-seq data) regulated by LPS in RAD21 KD MAC during LPS stimulation of control MAC. *P*-values in (**a**,**b**) were determined using two-sided rotation tests and BH corrected. **c** Bar plots showing expression levels of two cytokine genes typically activated during LPS-activation of MAC (controls: n=10 samples from five donors; LPS-treated: n=6 samples from three donors). **d**,**e** Comparison of transcription changes upon cohesin loss between human MAC with published murine BMM data[1]. **d** Heatmap presenting scaled human expression data of differentially LPS-induced genes (as shown in Figure 9c) along with independently scaled expression data of orthologous murine genes from LPS treated wildtype or RAD21-/- BMM. E Reproduction of the original clustering of genes provided in Figure 2d of Cuartero et al.[1] along with independently scaled expression data of orthologous human genes generated in this study. **f** Bar plots showing murine expression levels of genes (mean normalized RPKM ± SE based on the RNA-seq data) presented in Figure 9d and in **c** (*Ccl1* and *Ccl19* were not significantly expressed in BMM and not shown; n=3 independent experiments). (c-f) Source data are provided as a Source Data file.

Supplementary Table 1
Overlap between loop domain anchors and regions of open chromatin in MO and MO-derived cells

		Differentially accessible regions		Not differentially accessible regions				
**Comparison**	**More accessible in**	**Overlap with loop anchor**	**No overlap with loop anchor**	**Overlap with loop anchor**	**No overlap with loop anchor**	***P* value[1]**	**Odds ratio**	**Enriched motifs in loop anchor overlapping regions[2]**
moDC vs MO	MO	78	341	30900	80011	1.48×10^{-05}	0.59	*n.s.*
moDC vs MO	moDC	296	1460	30682	78892	2.45×10^{-27}	0.52	*n.s.*
MAC vs MO	MO	594	2114	34071	94050	3.25×10^{-08}	0.78	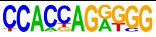 [3]
MAC vs MO	MAC	481	2361	34184	93803	2.36×10^{-34}	0.56	*n.s.*
moDC vs MAC	MAC	589	2574	36395	105012	8.12×10^{-21}	0.66	*n.s.*
moDC vs MAC	moDC	214	1071	36770	106515	1.63×10^{-14}	0.58	*n.s.*

[1]Fisher's exact test
[2]No motifs were found enriched in regions that were not overlapping compared to overlapping regions
[3]*De novo* derived motif for CTCF ($P < 10^{-13}$; target regions: 9.4%; background regions: 2.1%, hypergeometric test)

Supplementary Table 2
Overlap between loop domain anchors and H3K27ac-marked regions in MO and MO-derived cells

		Differential H3K27ac signal		No differential H3K27ac signal				
**Comparison**	**More H3K27ac in**	**Overlap with loop anchor**	**No overlap with loop anchor**	**Overlap with loop anchor**	**No overlap with loop anchor**	***P* value[1]**	**Odds ratio**	**Enriched motifs in loop anchor overlapping regions[2]**
moDC vs MO	MO	379	1114	10824	25708	0.00041	0.81	*n.s.*
moDC vs MO	moDC	400	1529	10803	25293	5.68×10^{-19}	0.61	*n.s.*
MAC vs MO	MO	674	1926	11760	26562	2.47×10^{-07}	0.79	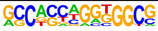 [3]
MAC vs MO	MAC	483	1778	11951	26710	4.57×10^{-23}	0.61	*n.s.*
moDC vs MAC	MAC	564	1816	11243	29464	2.55×10^{-05}	0.81	*n.s.*
moDC vs MAC	moDC	272	1014	11535	30266	1.85×10^{-07}	0.70	*n.s.*

[1]Fisher's exact test
[2]No motifs were found enriched in regions that were not overlapping compared to overlapping regions
[3]*De novo* derived motif for CTCF ($P < 10^{-11}$; target regions: 8.3%; background regions: 2.1% hypergeometric test)

Supplementary Table 3
Overlap between loop domain anchors and regions of open chromatin in siRNA treated cells

		**Differentially accessible regions**		**Not differentially accessible regions**				
**Comparison**	**More accessible in**	**overlap with loop anchor**	**no overlap with loop anchor**	**overlap with loop anchor**	**no overlap with loop anchor**	***P* value[1]**	**Odds Ratio**	**Enriched motifs in loop anchor overlapping regions[2]**
MAC RAD21 vs CTRL	CTRL	242	807	32876	100822	0.26	0.92	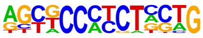 [3]
MAC RAD21 vs CTRL	RAD21 KD	54	266	33064	101363	0.0012	0.62	*n.s.*
MAC CTCF vs CTRL	CTRL	503	381	34372	104481	5.05×10^{-91}	4.01	*n.s.*
MAC CTCF vs CTRL	CTCF KD	21	99	34854	104763	0.072	0.64	*n.s.*
moDC RAD21 vs CTRL	CTRL	28	81	30468	99183	0.58	1.13	*n.s.*
moDC RAD21 vs CTRL	RAD21 KD	17	46	30479	99218	0.56	1.20	*n.s.*
moDC CTCF vs CTRL	CTRL	363	365	29386	86462	3.97×10^{-45}	2.93	*n.s.*
moDC CTCF vs CTRL	CTCF_KD	4	40	29745	86787	0.0092	0.29	*n.s.*

[1]Fisher's exact test
[2]No motifs were found enriched in regions that were not overlapping compared to overlapping regions
[3]*De novo* derived motif for CTCF ($P < 10^{-17}$; target regions: 27.0%; background regions: 5.3%, hypergeometric test)

Supplementary Table 4
Overlap between loop domain anchors and H3K27ac-marked regions in siRNA treated cells

Comparison	More H3K27ac in	Differential H3K27ac signal		No differential H3K27ac signal		*P* value[1]	Odds Ratio	Enriched motifs in loop anchor overlapping regions[2]
		overlap with loop anchor	no overlap with loop anchor	overlap with loop anchor	no overlap with loop anchor			
MAC RAD21 vs CTRL	CTRL	309	682	10421	27381	0.014	1.19	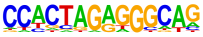 [3]
MAC RAD21 vs CTRL	RAD21 KD	537	1479	10193	26584	0.31	0.95	*n.s.*
MAC CTCF vs CTRL	CTRL	47	66	11244	27973	0.0034	1.77	*n.s.*
MAC CTCF vs CTRL	CTCF KD	44	81	11247	27958	0.12	1.35	*n.s.*
moDC RAD21 vs CTRL	CTRL	289	683	7828	25742	5.74×10^{-06}	1.39	*n.s.*
moDC RAD21 vs CTRL	RAD21 KD	105	391	8012	26034	0.25	0.87	*n.s.*
moDC CTCF vs CTRL	CTRL	21	21	9287	25990	0.0013	2.80	*n.s.*
moDC CTCF vs CTRL	CTCF_KD	4	9	9304	26002	0.76	1.24	*n.s.*

[1]Fisher's exact test
[2]No motifs were found enriched in regions that were not overlapping compared to overlapping regions
[3]*De novo* derived motif for CTCF ($P < 10^{-11}$; target regions: 9.8%; background regions: 0.7%, hypergeometric test)

Supplementary Table 5
siRNAs sequences

siRNA	Strand	Sequence (5‘--3‘)[1]
siCTRL	Sense	cuuAcGcuGAGuAcuucGAdTsdT
	Antisense	UCGAAGuACUcAGCGuAAGdTsdT
siRAD21_467 (a)	Sense	uacuGGGAGuAGuucGAAudTsdT
	Antisense	AUUCGAACuACUCCcAGuAdTsdT
siRAD21_2031 (b)	Sense	gcuuGAGuuAuGucGAAAudTsdT
	Antisense	AUUUCGAcAuAACUcAAGCdTsdT
siCTCF_3444 (a)	Sense	aguuAuGAuuucccAucGAdTsdT
	Antisense	UCGAUGGGAAAUcAuAACUdTsdT
siCTCF_3754 (b)	Sense	ccAucAGuuAGcuaucAGAdTsdT
	Antisense	UCUGAuAGCuAACUGAUGGdTsdT

[1]Chemical modification pattern:
A,G,U,C: RNA Nucleotide
a,g,u,c: 2´-O-Methyl-Nucleotide
dT: desoxy-T residue
s: Phosphorothioate

Supplementary Table 6
Hi-C-sequencing data generated in this study
(primary samples, accession IDs: EGAD00001007955, E-MTAB-10848)

Cell type	Sample	Donor	Total read pairs[1]
MO	freshly isolated	donor A	345600307
MO	freshly isolated	donor A	343912131
MAC	siCTCF.a transfected	donor A	526975706
MAC	siCTCF.b transfected	donor A	519101107
MAC	siCTRL transfected	donor A	488658410
MAC	mock transfected	donor A	461116051
MAC	siRAD21.a transfected	donor A	500289534
MAC	siRAD21.b transfected	donor A	488927759
moDC	siCTCF.a transfected	donor A	498460052
moDC	siCTCF.b transfected	donor A	486837233
moDC	siCTRL transfected	donor A	497191599
moDC	mock transfected	donor A	508011093
moDC	siRAD21.a transfected	donor A	460083285
moDC	siRAD21.b transfected	donor A	508542637
MO	freshly isolated	donor B	223671005
MO	freshly isolated	donor B	235214620
MAC	siCTCF.a transfected	donor B	280093949
MAC	siCTCF.b transfected	donor B	261370676
MAC	siCTRL transfected	donor B	189170392
MAC	mock transfected	donor B	269126657
MAC	siRAD21.a transfected	donor B	249068285
MAC	siRAD21.b transfected	donor B	267613603
moDC	siCTCF.a transfected	donor B	279146272
moDC	siCTCF.b transfected	donor B	269209771
moDC	siCTRL transfected	donor B	190228146
moDC	mock transfected	donor B	248570050
moDC	siRAD21.a transfected	donor B	254127896
moDC	siRAD21.b transfected	donor B	277914708
MO	freshly isolated	donor C	359637592
MO	freshly isolated	donor C	239358358
MAC	siCTCF.a transfected	donor C	331754511
MAC	siCTCF.b transfected	donor C	304643138
MAC	siCTRL transfected	donor C	299400088
MAC	mock transfected	donor C	272151827
MAC	siRAD21.a transfected	donor C	245435285
MAC	siRAD21.b transfected	donor C	324056188
moDC	siCTCF.a transfected	donor C	296348479
moDC	siCTCF.b transfected	donor C	312450654
moDC	siCTRL transfected	donor C	283578480
moDC	mock transfected	donor C	247008763
moDC	siRAD21.a transfected	donor C	234625539
moDC	siRAD21.b transfected	donor C	291207706

[1]Total raw read pairs after sequencing

Supplementary Table 7
H3K27ac ChIP-sequencing data generated in this study
(primary samples, accession IDs: EGAD00001007954, E-MTAB-10849)

Cell type	Sample	IP	Donor	Total reads[1]	FRIP[2]	Peaks[3]
MO	freshly isolated	H3K27ac ChIP	donor A	39093478	42.06%	32142
MO	freshly isolated	Input	donor A	23409999	–	–
MAC	siCTCF.a transfected	H3K27ac ChIP	donor A	22672977	47.99%	43968
MAC	siCTCF.b transfected	H3K27ac ChIP	donor A	33028014	55.20%	49511
MAC	siCTRL transfected	H3K27ac ChIP	donor A	34038238	58.90%	47052
MAC	mock transfected	H3K27ac ChIP	donor A	28099328	48.42%	45746
MAC	siRAD21.a transfected	H3K27ac ChIP	donor A	23651248	33.74%	34940
MAC	siRAD21.b transfected	H3K27ac ChIP	donor A	23923483	53.03%	39981
moDC	siCTCF.a transfected	H3K27ac ChIP	donor A	37952438	49.41%	47920
moDC	siCTCF.b transfected	H3K27ac ChIP	donor A	17708541	37.58%	40935
moDC	siCTRL transfected	H3K27ac ChIP	donor A	20889034	51.71%	45648
moDC	mock transfected	H3K27ac ChIP	donor A	18771884	57.25%	43758
moDC	siRAD21.a transfected	H3K27ac ChIP	donor A	35179505	36.12%	40041
moDC	siRAD21.b transfected	H3K27ac ChIP	donor A	15974146	38.60%	41877
MO	freshly isolated	H3K27ac ChIP	donor B	24080216	68.53%	35185
MO	freshly isolated	Input	donor B	26493459	–	–
MAC	siCTCF.a transfected	H3K27ac ChIP	donor B	16680247	69.10%	47268
MAC	siCTCF.b transfected	H3K27ac ChIP	donor B	19064988	56.47%	46074
MAC	siCTRL transfected	H3K27ac ChIP	donor B	21916076	69.38%	46418
MAC	mock transfected	H3K27ac ChIP	donor B	19913668	55.29%	41807
MAC	siRAD21.a transfected	H3K27ac ChIP	donor B	19577962	59.91%	39070
MAC	siRAD21.b transfected	H3K27ac ChIP	donor B	19832091	65.40%	39974
moDC	siCTCF.a transfected	H3K27ac ChIP	donor B	15926057	53.37%	41284
moDC	siCTCF.b transfected	H3K27ac ChIP	donor B	18667916	37.66%	37603
moDC	siCTRL transfected	H3K27ac ChIP	donor B	18860957	46.15%	40988
moDC	mock transfected	H3K27ac ChIP	donor B	18324157	42.85%	33074
moDC	siRAD21.a transfected	H3K27ac ChIP	donor B	15843354	43.16%	38053
moDC	siRAD21.b transfected	H3K27ac ChIP	donor B	16096400	36.92%	33631
MO	freshly isolated	H3K27ac ChIP	donor C	22864169	67.88%	31948
MO	freshly isolated	Input	donor C	25852225	–	–
MAC	siCTCF.a transfected	H3K27ac ChIP	donor C	20580228	54.11%	46691
MAC	siCTCF.b transfected	H3K27ac ChIP	donor C	41608005	46.16%	44711
MAC	siCTRL transfected	H3K27ac ChIP	donor C	21739371	47.32%	46101
MAC	mock transfected	H3K27ac ChIP	donor C	21535919	47.68%	44044
MAC	siRAD21.a transfected	H3K27ac ChIP	donor C	20020898	59.41%	41905
MAC	siRAD21.b transfected	H3K27ac ChIP	donor C	18464974	63.21%	41439
moDC	siCTCF.a transfected	H3K27ac ChIP	donor C	22829276	32.98%	39950
moDC	siCTCF.b transfected	H3K27ac ChIP	donor C	20211490	34.04%	37258
moDC	siCTRL transfected	H3K27ac ChIP	donor C	22812626	45.62%	43211
moDC	mock transfected	H3K27ac ChIP	donor C	21614248	30.92%	38298
moDC	siRAD21.a transfected	H3K27ac ChIP	donor C	16926661	56.54%	37616
moDC	siRAD21.b transfected	H3K27ac ChIP	donor C	23614317	50.45%	37800

[1]Unique reads after mapping to human reference genome GRCh38
[2]Fraction of reads in peaks (FRIP), determined by running HOMER's findPeaks program in "histone" mode using default parameters and the matching background (input)
[3]Number of peaks (determined by HOMER's findPeaks program with parameters "-style histone" and the matching donor background (input)

Supplementary Table 8
RAD21 ChIP-sequencing data generated in this study
(primary samples, accession IDs: EGAD00001007954, E-MTAB-10849)

Cell type	Sample	IP	Donor	Total reads[1]	FRIP[2]	Peaks[3]
MO	freshly isolated	RAD21 ChIP	donor A	15968487	9.60%	33181
MAC	siCTCF.a transfected	RAD21 ChIP	donor A	28858576	2.50%	16193
MAC	siCTCF.b transfected	RAD21 ChIP	donor A	27013842	2.11%	13658
MAC	siCTRL transfected	RAD21 ChIP	donor A	35808514	2.36%	16652
MAC	mock transfected	RAD21 ChIP	donor A	16626219	4.97%	21235
MAC	siRAD21.a transfected	RAD21 ChIP	donor A	13344413	4.47%	17596
MAC	siRAD21.b transfected	RAD21 ChIP	donor A	23860683	3.17%	14757
moDC	siCTCF.a transfected	RAD21 ChIP	donor A	23840644	1.14%	11001
moDC	siCTCF.b transfected	RAD21 ChIP	donor A	19323087	1.88%	15635
moDC	siCTRL transfected	RAD21 ChIP	donor A	20861381	2.83%	22211
moDC	mock transfected	RAD21 ChIP	donor A	16671143	3.21%	20961
moDC	siRAD21.a transfected	RAD21 ChIP	donor A	16416019	1.30%	10261
moDC	siRAD21.b transfected	RAD21 ChIP	donor A	21904089	0.91%	9745
MO	freshly isolated	RAD21 ChIP	donor B	27825476	7.90%	31435
MAC	siCTCF.a transfected	RAD21 ChIP	donor B	22069355	3.36%	15535
MAC	siCTCF.b transfected	RAD21 ChIP	donor B	20091949	8.20%	30878
MAC	siCTRL transfected	RAD21 ChIP	donor B	25394071	6.71%	27955
MAC	mock transfected	RAD21 ChIP	donor B	31723388	7.49%	31477
MAC	siRAD21.a transfected	RAD21 ChIP	donor B	18438103	3.02%	14965
MAC	siRAD21.b transfected	RAD21 ChIP	donor B	19432620	4.72%	19835
moDC	siCTCF.a transfected	RAD21 ChIP	donor B	17111363	1.75%	11914
moDC	siCTCF.b transfected	RAD21 ChIP	donor B	15657639	1.12%	9885
moDC	siCTRL transfected	RAD21 ChIP	donor B	16181161	1.36%	11714
moDC	mock transfected	RAD21 ChIP	donor B	13713128	2.46%	14831
moDC	siRAD21.a transfected	RAD21 ChIP	donor B	13823379	0.69%	5710
moDC	siRAD21.b transfected	RAD21 ChIP	donor B	14071650	1.81%	11530
MO	freshly isolated	RAD21 ChIP	donor C	20461050	4.18%	23224
MAC	siCTCF.a transfected	RAD21 ChIP	donor C	18823401	0.38%	3622
MAC	siCTCF.b transfected	RAD21 ChIP	donor C	19021628	1.09%	8230
MAC	siCTRL transfected	RAD21 ChIP	donor C	21192537	0.78%	7380
MAC	mock transfected	RAD21 ChIP	donor C	23771329	2.30%	15728
MAC	siRAD21.a transfected	RAD21 ChIP	donor C	14686297	0.33%	2813
MAC	siRAD21.b transfected	RAD21 ChIP	donor C	16073344	0.77%	5339
moDC	siCTCF.a transfected	RAD21 ChIP	donor C	16914213	0.87%	6679
moDC	siCTCF.b transfected	RAD21 ChIP	donor C	14215046	6.24%	27873
moDC	siCTRL transfected	RAD21 ChIP	donor C	19858998	0.88%	7989
moDC	mock transfected	RAD21 ChIP	donor C	18950198	9.88%	44638
moDC	siRAD21.a transfected	RAD21 ChIP	donor C	16821169	0.23%	2134
moDC	siRAD21.b transfected	RAD21 ChIP	donor C	19111129	0.36%	3132

[1]Unique reads after mapping to human reference genome GRCh38
[2]Fraction of reads in peaks (FRIP), determined by running HOMER's findPeaks program in ''factor'' mode using default parameters and the matching background (input)
[3]Number of peaks (determined by HOMER's findPeaks program with parameters "-style factor" and the matching donor background (input)

Supplementary Table 9
CTCF ChIP-sequencing data generated in this study
(primary samples, accession IDs: EGAD00001007954, E-MTAB-10849)

Cell type	Sample	IP	Donor	Total reads[1]	FRIP[2]	Peaks[3]
MO	freshly isolated	CTCF ChIP	donor A	4772887	12.03%	22079
MAC	siCTCF.a transfected	CTCF ChIP	donor A	25050706	0.72%	6150
MAC	siCTCF.b transfected	CTCF ChIP	donor A	27734197	1.73%	13140
MAC	siCTRL transfected	CTCF ChIP	donor A	30448840	2.29%	18622
MAC	mock transfected	CTCF ChIP	donor A	13005407	6.78%	24319
MAC	siRAD21.a transfected	CTCF ChIP	donor A	13100939	4.10%	18592
MAC	siRAD21.b transfected	CTCF ChIP	donor A	27447089	4.20%	22333
moDC	siCTCF.a transfected	CTCF ChIP	donor A	17877544	1.73%	11240
moDC	siCTCF.b transfected	CTCF ChIP	donor A	19381861	1.52%	9368
moDC	siCTRL transfected	CTCF ChIP	donor A	14777094	0.96%	7128
moDC	mock transfected	CTCF ChIP	donor A	9134267	3.59%	15221
moDC	siRAD21.a transfected	CTCF ChIP	donor A	18743627	0.92%	8305
moDC	siRAD21.b transfected	CTCF ChIP	donor A	12260087	2.11%	12695
MO	freshly isolated	CTCF ChIP	donor B	31643245	16.24%	33247
MAC	siCTCF.a transfected	CTCF ChIP	donor B	22264016	0.83%	7031
MAC	siCTCF.b transfected	CTCF ChIP	donor B	22566422	3.46%	16089
MAC	siCTRL transfected	CTCF ChIP	donor B	26450317	0.59%	7271
MAC	mock transfected	CTCF ChIP	donor B	25887048	7.93%	28498
MAC	siRAD21.a transfected	CTCF ChIP	donor B	20931766	1.73%	14740
MAC	siRAD21.b transfected	CTCF ChIP	donor B	17460350	4.33%	21567
moDC	siCTCF.a transfected	CTCF ChIP	donor B	13484471	0.81%	6210
moDC	siCTCF.b transfected	CTCF ChIP	donor B	12759379	0.15%	1151
moDC	siCTRL transfected	CTCF ChIP	donor B	13173047	2.10%	15111
moDC	mock transfected	CTCF ChIP	donor B	16289487	0.22%	2345
moDC[4]	siRAD21.a transfected	CTCF ChIP	donor B	15173899	0.05%	303
moDC	siRAD21.b transfected	CTCF ChIP	donor B	14372072	0.64%	5927
MO	freshly isolated	CTCF ChIP	donor C	21574796	5.07%	22808
MAC[4]	siCTCF.a transfected	CTCF ChIP	donor C	17103474	0.01%	61
MAC	siCTCF.b transfected	CTCF ChIP	donor C	12264789	3.66%	16387
MAC	siCTRL transfected	CTCF ChIP	donor C	22021415	3.42%	20600
MAC	mock transfected	CTCF ChIP	donor C	19253786	2.19%	16959
MAC	siRAD21.a transfected	CTCF ChIP	donor C	14335132	5.09%	21958
MAC	siRAD21.b transfected	CTCF ChIP	donor C	16213003	4.23%	20071
moDC	siCTCF.a transfected	CTCF ChIP	donor C	22294662	1.62%	9883
moDC	siCTCF.b transfected	CTCF ChIP	donor C	27314323	5.56%	20285
moDC	siCTRL transfected	CTCF ChIP	donor C	12861266	5.10%	23539
moDC	mock transfected	CTCF ChIP	donor C	11263762	9.81%	27637
moDC	siRAD21.a transfected	CTCF ChIP	donor C	11403520	6.05%	22628
moDC	siRAD21.b transfected	CTCF ChIP	donor C	19370219	4.03%	22624

[1]Unique reads after mapping to human reference genome GRCh38
[2]Fraction of reads in peaks (FRIP), determined by running HOMER's findPeaks program in "factor" mode using default parameters and the matching background (input)
[3]Number of peaks (determined by HOMER's findPeaks program with parameters "-style factor" and the matching donor background (input)
[4]Low quality samples excluded from analysis

Supplementary Table 10
ATAC-sequencing data generated in this study
(primary samples, accession IDs: EGAD00001007953, E-MTAB-10846)

Cell type	Sample	donor	Total read pairs[1]	FRIP (%)[2]	Peaks[3]
MO	freshly isolated	donor A	43698326	9.79%	51987
MAC	mock transfected	donor A	26373907	47.58%	118312
MAC	siCTRL transfected	donor A	36248496	39.26%	114793
MAC	siCTCF.a transfected	donor A	42968894	50.55%	130417
MAC	siCTCF.b transfected	donor A	31374411	56.97%	134462
MAC	siRAD21.a transfected	donor A	29509727	28.67%	72053
MAC	siRAD21.b transfected	donor A	31295575	43.38%	113101
moDC	mock transfected	donor A	37511535	22.65%	63730
moDC[4]	siCTRL transfected	donor A	33171645	7.78%	5255
moDC	siCTCF.a transfected	donor A	30790880	28.68%	69038
moDC	siCTCF.b transfected	donor A	39920787	23.27%	36760
moDC	siRAD21.a transfected	donor A	27551103	40.28%	47764
moDC	siRAD21.b transfected	donor A	33590272	30.90%	51145
MO	freshly isolated	donor B	26431271	7.56%	35244
MAC	mock transfected	donor B	38239563	69.36%	147122
MAC	siCTRL transfected	donor B	36038752	62.43%	137881
MAC	siCTCF.a transfected	donor B	37098311	63.27%	135132
MAC	siCTCF.b transfected	donor B	30281617	58.65%	133692
MAC	siRAD21.a transfected	donor B	33571785	45.46%	115104
MAC	siRAD21.b transfected	donor B	31149256	51.40%	119118
moDC	mock transfected	donor B	23837099	46.27%	115629
moDC	siCTRL transfected	donor B	21296934	12.53%	17232
moDC	siCTCF.a transfected	donor B	29687021	29.13%	95621
moDC	siCTCF.b transfected	donor B	29157045	13.03%	17554
moDC	siRAD21.a transfected	donor B	29069416	30.40%	29129
moDC	siRAD21.b transfected	donor B	28018638	14.44%	26412
MO	freshly isolated	donor C	25817047	9.89%	38973
MAC	mock transfected	donor C	28732576	10.67%	47531
MAC	siCTRL transfected	donor C	36631756	20.35%	74209
MAC	siCTCF.a transfected	donor C	32076289	9.67%	49219
MAC	siCTCF.b transfected	donor C	36328365	48.52%	114002
MAC	siRAD21.a transfected	donor C	31474186	13.85%	60407
MAC	siRAD21.b transfected	donor C	33189796	11.19%	53651
moDC	mock transfected	donor C	26033471	20.66%	75407
moDC	siCTRL transfected	donor C	26604757	30.55%	102985
moDC	siCTCF.a transfected	donor C	30388188	21.97%	86734
moDC	siCTCF.b transfected	donor C	24835173	9.03%	42761
moDC	siRAD21.a transfected	donor C	34210196	51.82%	142393
moDC	siRAD21.b transfected	donor C	33234893	48.36%	139762

[1]Unique read pairs after mapping to human reference genome GRCh38
[2]Fraction of reads in peaks (FRIP), determined by running HOMER’s findPeaks with parameters "-region -size 150"
[3]Number of peaks (determined by HOMER’s findPeaks program with parameters "-region -size 150"
[4]Low quality sample excluded from analysis

Supplementary Table 11
RNA-sequencing data generated in this study
(primary samples, accession IDs: EGAD00001007956, E-MTAB-10844)

Cell type	Sample	Donor	RIN[1]	Total reads	Uniquely Mapped Reads (%)[2]
MO	freshly isolated	donor A	10.0	38148355	64.48%
MAC	siCTRL transfected	donor A	9.7	40290249	89.23%
MAC	siCTCF.a transfected	donor A	9.6	38670860	92.36%
MAC	siCTCF.b transfected	donor A	9.5	45600139	90.60%
MAC	siRAD21.a transfected	donor A	9.6	45081029	86.29%
MAC	siRAD21.b transfected	donor A	9.4	41220979	89.46%
moDC	siCTRL transfected	donor A	9.1	39010783	67.43%
moDC	siCTCF.a transfected	donor A	9.5	40299215	90.45%
moDC	siCTCF.b transfected	donor A	8.9	36217788	77.29%
moDC	siRAD21.a transfected	donor A	8.9	40132364	84.76%
moDC	siRAD21.b transfected	donor A	9.0	36234369	77.73%
MO	freshly isolated	donor B	10.0	43797598	91.60%
MAC	siCTRL transfected	donor B	9.6	40171544	91.10%
MAC	siCTCF.a transfected	donor B	9.5	35370682	90.15%
MAC	siCTCF.b transfected	donor B	9.6	40042520	88.31%
MAC	siRAD21.a transfected	donor B	9.5	41685358	38.11%
MAC	siRAD21.b transfected	donor B	9.6	34425591	85.08%
moDC	siCTRL transfected	donor B	9.1	35893686	90.38%
moDC	siCTCF.a transfected	donor B	9.8	38870032	91.00%
moDC	siCTCF.b transfected	donor B	9.2	39024554	78.52%
moDC	siRAD21.a transfected	donor B	9.1	38902543	83.20%
moDC	siRAD21.b transfected	donor B	9.0	46974124	88.53%
MO	freshly isolated	donor C	8.8	29590525	58.38%
MAC	siCTRL transfected	donor C	9.3	44613294	65.08%
MAC	siCTCF.a transfected	donor C	9.3	38768174	47.75%
MAC	siCTCF.b transfected	donor C	9.1	39372803	70.62%
MAC	siRAD21.a transfected	donor C	9.1	42299478	85.85%
MAC	siRAD21.b transfected	donor C	8.9	20132929	44.69%
moDC	siCTRL transfected	donor C	9.7	45504324	38.56%
moDC	siCTCF.a transfected	donor C	9.5	41178145	58.34%
moDC	siCTCF.b transfected	donor C	9.3	49683787	52.96%
moDC	siRAD21.a transfected	donor C	9.7	35602824	70.40%
moDC	siRAD21.b transfected	donor C	9.5	39038979	69.57%
MO	freshly isolated	donor D	9.2	29181075	81.32%
moDC	mock transfected	donor D	8.8	32755259	85.55%
moDC	siCTRL transfected	donor D	8.9	27880524	86.26%
moDC	siCTCF.a transfected	donor D	9.1	26699752	86.86%
moDC	siCTCF.b transfected	donor D	8.6	23848238	86.02%
moDC	siRAD21.a transfected	donor D	8.8	21165889	83.80%
moDC	siRAD21.b transfected	donor D	8.7	20802223	84.85%
moDC	siCTCF.a transfected	donor E	7.9	46414045	87.71%
moDC	siCTCF.b transfected	donor E	9.0	52530862	87.92%
moDC	siRAD21.a transfected	donor E	8.4	44429482	88.21%
moDC	siRAD21.b transfected	donor E	8.8	51994457	88.06%
MO	freshly isolated	donor F	9.8	41270923	90.74%
MAC	mock transfected	donor F	9.5	37009537	88.57%
MAC	siCTRL transfected	donor F	9.3	38742723	86.44%
MAC	siRAD21.a transfected	donor F	9.3	40971004	87.50%
MAC	siRAD21.b transfected	donor F	9.5	43451037	85.92%
MO	freshly isolated	donor G	9.9	23480003	91.05%
MAC	mock transfected	donor G	9.7	18438305	89.40%
MAC	siCTRL transfected	donor G	9.2	23318255	90.24%
MAC	siRAD21.a transfected	donor G	9.6	20003936	85.20%
MAC	siRAD21.b transfected	donor G	9.5	21729250	86.70%

[1]RNA-Integrity Index (RIN) as measured using the TapeStation (Agilent)
[2]percentage of unique sequences after mapping to GRCh38

Supplementary Table 12
RNA-sequencing data generated in this study (primary samples, LPS activated, accession IDs: EGAD00001007952, E-MTAB-10845)

Cell type	Sample	Treatment	Donor	RIN[1]	Total reads	Uniquely Mapped Reads (%)[2]
MAC	mock transfected	none, 4h	donor H	9.8	44198385	89.86%
MAC	siCTRL transfected	none, 4h	donor H	9.3	31442859	89.16%
MAC	siRAD21.a transfected	none, 4h	donor H	9.1	35181598	87.09%
MAC	siRAD21.b transfected	none, 4h	donor H	9.9	40373371	87.67%
MAC	mock transfected	LPS, 4h	donor H	10	51454905	88.62%
MAC	siCTRL transfected	LPS, 4h	donor H	9.8	43543298	88.57%
MAC	siRAD21.a transfected	LPS, 4h	donor H	9.8	58346740	89.75%
MAC	siRAD21.b transfected	LPS, 4h	donor H	9.9	46481863	88.63%
MAC	mock transfected	none, 4h	donor I	9.4	29404187	92.48%
MAC	siCTRL transfected	none, 4h	donor I	9	30613417	89.17%
MAC	siRAD21.a transfected	none, 4h	donor I	9.3	25692258	84.45%
MAC	siRAD21.b transfected	none, 4h	donor I	9.2	30243203	88.85%
MAC	mock transfected	LPS, 4h	donor I	9.8	32347633	90.52%
MAC	siCTRL transfected	LPS, 4h	donor I	9.4	33044363	88.70%
MAC	siRAD21.a transfected	LPS, 4h	donor I	8.4	27418010	88.96%
MAC	siRAD21.b transfected	LPS, 4h	donor I	9.3	30027812	89.84%
MAC	mock transfected	none, 4h	donor J	9.5	24069268	90.34%
MAC	siCTRL transfected	none, 4h	donor J	9.6	25567525	89.78%
MAC	siRAD21.a transfected	none, 4h	donor J	9.8	23231206	90.06%
MAC	siRAD21.b transfected	none, 4h	donor J	9.8	33544126	84.56%
MAC	mock transfected	LPS, 4h	donor J	9.8	33502358	89.50%
MAC	siCTRL transfected	LPS, 4h	donor J	9.7	26808300	90.81%
MAC	siRAD21.a transfected	LPS, 4h	donor J	10	25194505	90.16%
MAC	siRAD21.b transfected	LPS, 4h	donor J	9.9	26761290	89.72%

[1]RNA-Integrity Index (RIN) as measured using the TapeStation (Agilent)
[2]percentage of unique sequences after mapping to GRCh38

Supplementary Table 13
Published human CAGE-sequencing data
(NCBI BioProject accession ID: PRJDB1099)

Cell type	Sample name	Donor	Total reads	EGA sample ID	Reference
$CD14^{++}CD16^{-}$ MO	CNhs13216	donor A	37296719	DRR009038	[2]
$CD14^{++}CD16^{-}$ MO	CNhs13224	donor B	42312430	DRR009037	„
$CD14^{++}CD16^{-}$ MO	CNhs13540	donor C	60840438	DRR009039	„
moDC	CNhs10855	donor D	18921543	DRR009084	„
moDC	CNhs11062	donor E	45270915	DRR009085	„
moDC	CNhs12195	donor F	15120229	DRR008944	„
moDC	CNhs12000	donor G	39973367	DRR009086	„
MAC	CNhs10861	donor H	21746594	DRR009194	„
MAC	CNhs11899	donor I	44715811	DRR009195	„
MAC	CNhs12003	donor J	31379422	DRR009196	„

Supplementary Table 14
Published human RNA-sequencing data
(accession IDs: EGAD00001006604, E-MTAB-9929)

Cell type	Sample	Donor	RIN[1]	Total reads	Uniquely Mapped Reads[2]	EGA sample ID	Reference
MO	freshly isolated	donor A	10.0	38148355	64.48%	EGAN00002795366	[3]
MAC	mock transfected	donor A	9.9	40479286	90.04%	EGAN00002795358	„
moDC	mock transfected	donor A	9	42986973	82.06%	EGAN00002795376	„
MO	freshly isolated	donor B	10.0	43797598	91.60%	EGAN00002795367	„
MAC	mock transfected	donor B	9.4	42061245	91.22%	EGAN00002795359	„
moDC	mock transfected	donor B	9.6	37261048	90.16%	EGAN00002795413	„
MO	freshly isolated	donor C	8.8	29590525	58.38%	EGAN00002795368	„
MAC	mock transfected	donor C	9.5	43159887	49.02%	EGAN00002795360	„
moDC	mock transfected	donor C	9.2	46745415	68.07%	EGAN00002795377	„
MO	freshly isolated	donor E	9.3	53456066	85.60%	EGAN00002795363	„
moDC	mock transfected	donor E	9.2	39468070	88.30%	EGAN00002795373	„
moDC	siCTRL transfected	donor E	9.1	44102490	87.33%	EGAN00002795378	„

[1]RNA-Integrity Index (RIN) as measured using the TapeStation (Agilent)
[2]percentage of unique sequences after mapping to GRCh38

Supplementary Table 15
Published murine RNA-sequencing data
(GEO accession ID: GSE108599)

Cell type	Sample	Replicate	Total reads	Uniquely Mapped Reads[1]	GEO sample ID	SRA sample ID	Reference
BMM	WT_unstimulated	rep1	56930174	85.86%	GSM2905510	SRR6492159	[1]
BMM	WT_unstimulated	rep2	76200359	86.02%	GSM2905511	SRR6492160	„
BMM	WT_unstimulated	rep3	74064911	86.91%	GSM2905512	SRR6492161	„
BMM	WT_LPS_2h	rep1	61463786	84.97%	GSM2905513	SRR6492162	„
BMM	WT_LPS_2h	rep2	53015330	85.94%	GSM2905514	SRR6492163	„
BMM	WT_LPS_2h	rep3	85721517	88.00%	GSM2905515	SRR6492164	„
BMM	WT_LPS_8h	rep1	57381792	84.05%	GSM2905516	SRR6492165	„
BMM	WT_LPS_8h	rep2	48679830	86.65%	GSM2905517	SRR6492166	„
BMM	WT_LPS_8h	rep3	85011435	90.02%	GSM2905518	SRR6492167	„
BMM	Rad21-/-_unstimulated	rep1	74966709	86.18%	GSM2905519	SRR6492168	„
BMM	Rad21-/-_unstimulated	rep2	62815855	86.48%	GSM2905520	SRR6492169	„
BMM	Rad21-/-_unstimulated	rep3	94722799	80.47%	GSM2905521	SRR6492170	„
BMM	Rad21-/-_LPS_2h	rep1	72710489	84.11%	GSM2905522	SRR6492171	„
BMM	Rad21-/-_LPS_2h	rep2	57239032	86.03%	GSM2905523	SRR6492172	„
BMM	Rad21-/-_LPS_2h	rep3	80493811	88.71%	GSM2905524	SRR6492173	„
BMM	Rad21-/-_LPS_8h	rep1	66069872	83.53%	GSM2905525	SRR6492174	„
BMM	Rad21-/-_LPS_8h	rep2	66554311	86.30%	GSM2905526	SRR6492175	„
BMM	Rad21-/-_LPS_8h	rep3	89334607	85.90%	GSM2905527	SRR6492176	„

[1]percentage of unique sequences after mapping to GRCm38

Supplementary References

1. Cuartero S*, et al.* Control of inducible gene expression links cohesin to hematopoietic progenitor self-renewal and differentiation. *Nat Immunol* **19**, 932-941 (2018).

2. Consortium F*, et al.* A promoter-level mammalian expression atlas. *Nature* **507**, 462-470 (2014).

3. Mendes K*, et al.* The epigenetic pioneer EGR2 initiates DNA demethylation in differentiating monocytes at both stable and transient binding sites. *Nat Commun* **12**, 1556 (2021).
