# Supplementary material for: Postmitotic differentiation of human monocytes requires cohesin-structured chromatin
Source: Nat Commun. 2022 Jul 25;13:4301. doi: 10.1038/s41467-022-31892-2 (PMC9314343; doi:10.1038/s41467-022-31892-2)
Supplement: Supplementary file 3 — Description of Additional Supplementary Files [file 41467_2022_31892_MOESM3_ESM.docx]

**Description of Additional Supplementary Files**

**Supplementary Data File 1:** Peak positions and results of differential gene or peak analyses for MO, moDC and MAC.

**Supplementary Data File 2:** Positions and results of differential analyses of TADs and loops, as well as RAD21 and CTCF peaks for MO, moDC and MAC.

**Supplementary Data File 3:** Peak positions and results of differential gene or peak analyses for siRNA treated cells.

**Supplementary Data File 4:** Positions and results of differential analyses of TADs and loops, as well as RAD21 and CTCF peaks for siRNA treated cells.
